# Does Temperature Tolerance Increase in Long-Term Domesticated *Frankliniella occidentalis* Under Constant Temperature?

**DOI:** 10.3390/insects16060557

**Published:** 2025-05-24

**Authors:** Lin Shu, Hongbo Li, Yawen Chang, Yuzhou Du

**Affiliations:** 1 Institute of Applied Entomology, College of Plant Protection, Yangzhou University, Yangzhou 225009, China; mx120230848@stu.yzu.edu.cn (L.S.); changyawen@yzu.edu.cn (Y.C.); 2 Institute of Plant Protection, Guizhou Academy of Agricultural Sciences, Guiyang 550009, China; gzlhb2017@126.com; 3 Jiangsu Province Engineering Research Center of Green Pesticides, Yangzhou University, Yangzhou 225009, China

**Keywords:** *Frankliniella occidentalis*, population evolution, temperature adaptation, facultative parthenogenesis, arrhenotoky

## Abstract

The wide distribution of *Frankliniella occidentalis* is largely due to its extreme temperature adaptability. In current studies, most scholars consider environmental changes to be the main factor affecting insect temperature adaptation. However, our previous studies have shown that the adaptability of *F. occidentalis* to extreme temperature conditions can be strengthened through domestication. In this study, the population of *F. occidentalis* raised in the laboratory for a long time (2008–2022) under relatively constant temperature and humidity conditions was used as the experimental material. Over 14 years, changes in temperature tolerance after the same high- and low-temperature stress were evaluated by comparing the survival data of the 2010 population, 2016 population (more than 100 generations), and 2022 population (more than 200 generations). The survival data and LT_50_ values demonstrated significant stage- and sex-specific differences in thermal tolerance: The cold tolerance of *F. occidentalis* improved significantly, with LT_50_ decreasing from −12.5 °C (P2010) to −13.4 °C (P2022) for females and −11.5 °C to −13.0 °C for males. Notably, male adults showed higher survival rates than females at −14 °C and −15 °C. Meanwhile, heat tolerance increased most markedly in 2nd instar larvae (ΔLT50 = +4.1 °C). These findings indicate an environment-independent evolutionary pathway within the population, providing a new research direction for insect population evolution.

## Simple Summary

The wide distribution of *Frankliniella occidentalis* is largely due to its extreme temperature adaptability. In this study, a population of *F. occidentalis* raised in the laboratory for a long time (2008–2022) under relatively constant temperature and humidity conditions was used as the experimental material, and the high- and low-temperature tolerance of this population was measured several times over 14 years. The results showed that the heat and cold tolerance of *F. occidentalis* at all ages and sexes were different and increased with the extension of laboratory rearing time, indicating that there is an environment-independent evolutionary pathway within the population. Our results can provide a new research direction for insect population evolution.

## 1. Introduction

In nature, external environmental factors have a significant impact on individual insects [1], and the adaptability of insects to the external environment determines their survival, reproduction, population growth, and other biological processes and also directly relates to the geographical distribution of the insect population. The environmental adaptability of insects can be divided into heat resistance, cold resistance, drought resistance, moisture resistance, disease resistance, hunger resistance, and so on. Temperature is one of the most closely related environmental factors to insects. The strength of heat and cold tolerance of insects directly determines their growth, development, and population colonization.

At present, researchers generally believe that climate change drives insects to undergo behavioral and physiological adaptations, leading to increased tolerance in insect populations. In recent years, “acclimation” has gained significant attention in research. In many studies, acclimating insects to sublethal temperature stress for a period of time has been shown to improve their heat or cold tolerance. The exposure times insects may experience at different temperature challenges may range from many days or weeks (developmental acclimation) to a few days (acclimation) or just minutes or hours (hardening) [2]. When insects are exposed to temperature stress, other stressors may also interact. Boardman discussed the potential molecular mechanisms underlying crosstalk among these stressors [3]. One study found that by “acclimation”, *Drosophila melanogaster* can be bred to be a population that is nearly resistant to cold stress, thereby protecting the entire population [4]. Another study found continuous mild hypothermia to significantly improve both cold and heat resistance in *Frankliniella occidentalis* (Pergande) (Thysanoptera: Thripidae), western flower thrips (WFT), with a maximum increase in survivorship of cold temperatures being achieved by pretreating larvae or adults at 0 °C for 2 h [5,6]. It was also proved that the heat adaptability brought about by acclimation had a transgenerational effect. The heat tolerance acquired by *Cnaphalocrocis medinalis* through heat training can be inherited from parents to offspring, and the tolerant ability to high temperature can still be partially maintained in two generations after stopping heat acclimation [7]. In a manner of speaking, thermal acclimation has promoted the evolution of temperature tolerance in insect populations, but whether the evolution of thermal tolerance is only caused by external environmental factors has not been determined because of the complexity of insect evolution, and there may be another evolutionary mechanism within insect populations that is not induced by external environmental factors.

Western flower thrips is a globally significant economic pest, causing damage to numerous crops, including tomatoes, beans, and tobacco. The species, native to western North America, damages plants through direct feeding and the transmission of plant viruses [8]. In 2003, WFT was recorded in greenhouse pepper in Beijing for the first time [9]. Since then, WFT has been detected in 14 provinces across China [10]. In addition to its economic impact, the reproductive strategies of *F. occidentalis* have attracted scientific attention. This species mainly reproduces by means of both sexual reproduction and arrhenotoky. Sexual reproduction provides populations with new genotypic combinations under the action of natural selection and promotes the elimination of harmful mutations. Haplodiploidy caused by arrhenotoky can effectively reduce the genetic load, due to a lower effective mutation rate and the exposure of deleterious recessive alleles in haploid males, and increase the rate at which rare recessive beneficial mutations can spread [11]. The coexistence of the two reproductive modes could theoretically enhance the development and evolution of insect colonies, but there is a lack of relevant research to validate this hypothesis.

Therefore, based on the above hypotheses and questions, we conducted the following study: We maintained an *F. occidentalis* population under controlled laboratory conditions with constant temperature (26 ± 0.5 °C) and humidity for long-term rearing. Previous studies have established that *F. occidentalis* develops optimally between 25–30 °C [12], with the highest survival rates across all life stages observed at approximately 26 °C [13,14,15]. Accordingly, we set the temperature at this optimal 26 °C to minimize environmental stress and other confounding factors. Through a 14-year study (2008–2022), we systematically evaluated thermal tolerance evolution in laboratory-acclimated *F. occidentalis* populations. A comprehensive comparative analysis was conducted across three key generations—the 2010 population, the 2016 population (more than 100 generations), and the 2022 population (more than 200 generations)—evaluating critical thermal tolerance parameters, including survival rates under high- and low-temperature stress and lower and upper lethal temperatures (LT_50_) across different developmental stages (2nd instar larvae and adults) and sexes. Our results provide experimental support for the relationship between insect population evolution and reproductive mode, offer new insights into intrinsic evolutionary mechanisms beyond environmental selection, and suggest a new research direction for the study of insect population evolution.

## 2. Materials and Methods

### 2.1. Establishment of Insect Populations

The *Frankliniella occidentalis* used in our experiment were from a colony started with insects collected in Hangzhou, China in 2008 and reared in the laboratory as described by Li et al. [5]. The thrips life stages assessed in this experiment were 1-day-old 2nd instar larvae and 3-day-old adults (both sexes). In our experiments, the survival of thrips was assessed at high or low temperatures for each of three populations: all three populations were reared in a climate chamber (Shanghai, China) at a constant 26 ± 0.5 °C and a 16:8 h L:D photoperiod. The 2010 (P2010), 2016 (P2016), and 2022 populations (P2022) are the offspring of P2008 and no external sources were introduced during the experiment, and each population was separated by about 100 generations. Each population was then subjected to a high or low temperature for one hour and their survival rate determined.

### 2.2. Cold Exposure

The method of cold exposure in this study was the same as that of previous experiments [5]. In P2010, four replicates of 30 adult WFT of each sex and 2nd larvae were collected from rearing colonies and placed by group in 1.5 mL centrifuge tubes, which were placed in small glass tubes. Groups of WFT were then exposed to a particular temperature within a range of temperatures (from −8 °C to −14 °C with 1 °C increments) for 1 h. The target temperature was maintained by a temperature control (accuracy: ±0.5 °C) (DC-3010, Ningbo, China), which allowed for sub-zero temperature use. The experiment groups were allowed to recover in a chamber at 26 ± 0.5 °C. After 2 h, the survival of each WFT larva or adult was scored. An individual was considered to have survived if they could walk normally when prodded gently with a brush. All treatments were replicated four times.

In P2016 and P2022, we conducted a pre-test using the same methods of cold exposure as described above for P2010 [5]. This pre-test demonstrated that the initial lethal cold temperature for the P2016 and P2022 was −10 °C. Thus, groups of WFT were exposed to a particular temperature within a range of temperatures (from −15 °C to −10 °C with 1 °C increments, rather than from −8 °C to −14 °C).

### 2.3. Heat Exposure

The method of heat exposure in this study was similarly adapted from previous experiments [5]. In P2010, the treatment in which thrips were exposed to high temperature was similar to that described above for exposure to low temperatures. Groups of 30 WFT adults and 2nd instar larvae were exposed to different high temperatures (33 to 41 °C at 2 °C increments) for 1 h. The treatment and recovery time was the same as for the cold exposure experiment. Each treatment was replicated four times.

In P2016, the result of a pre-test showed the initial lethal heat temperature to be 37 °C, so we determined the range of exposure high temperatures was from 37 to 41 °C in 1 °C increments.

In P2022, the initial lethal heat temperature was same as P2016, but the range of exposure to high temperatures was changed to from 37 to 42 °C in 1 °C increments because of the pre-experimental results.

### 2.4. Statistical Analysis

Prior to data analysis, all datasets were tested for normality using the Shapiro–Wilk test and for homogeneity of variance using Levene’s test. For data that failed to meet the assumptions of normality or homogeneity of variance, appropriate data transformations (e.g., logarithmic transformation) were applied to satisfy the prerequisites for ANOVA. Differences between treatments were analyzed either by *t*-test or by one-way ANOVA analysis of variance. Multiple comparisons of survival among development stages, sex, and their combined effect were determined by a least significant difference (LSD) test (homogeneous variances) or Dunnett’s C test (non-homogeneous variances) with *p* < 0.05. Probit regression was used to estimate values of the temperature causing 50% of tested individuals to die in one hour (LT_50_). Data were analyzed using SPSS 16.0 software [16].

## 3. Results

### 3.1. Cold Exposure

**Figure 1:**
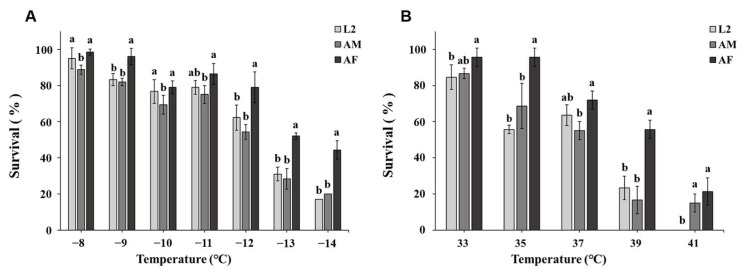
Effect of temperature shock on survival rate of *F. occidentalis* in 2010. Data are denoted as means ± SD. Different lowercase letters indicate significant differences among developmental stages within the same temperature (*p* < 0.05). AF: adult female, AM: adult male, L2: 2nd instar larvae. (**A**) Low-temperature treatments. (**B**) High-temperature treatments.

Low-temperature shocks, sex/stage, and the interaction of temperature × sex/stage all had significant effects on the survival of P2010 [5] (temperature: *F* = 119.86, *df* = 6, *p* < 0.001; sex/stage: *F* = 26.07, *df* = 2, *p* < 0.001). However, we found no significant effect for the interaction of temperature × sex (*F* = 1.47, *df* = 12, *p* = 0.153). After exposure to a range of low temperatures, the decline in the survival of females was slower than that of males and 2nd instar larvae. When exposed to −14 °C, female survival remained 42%, clearly demonstrating females to be more tolerant of low temperatures. Survival of males was 89, 81, 76, 71, 52, 31, and 23% at −8, −9, −10, −11, −12, −13, and −14 °C, respectively. For these temperatures, survival of 2nd instar larvae was 95, 86, 81, 77, 66, 34, and 19%, respectively. The initial lethal temperature of WFT was close to −8 °C (−8 °C: *F*_2,6_ =32.416, *p* = 0.001; −9 °C: *F*_2,6_ = 14.834, *p* = 0.005; −10 °C: *F*_2,6_ = 12.774, *p* = 0.014; −11 °C: *F*_2,6_ = 4.095, *p* = 0.046; −12 °C: *F*_2,6_ = 10.401, *p* = 0.011; −13 °C: *F*_2,6_ = 30.112, *p* = 0.001; −14 °C: *F*_2,6_ = 76.797, *p* < 0.001) (Figure 1A).

**Figure 2:**
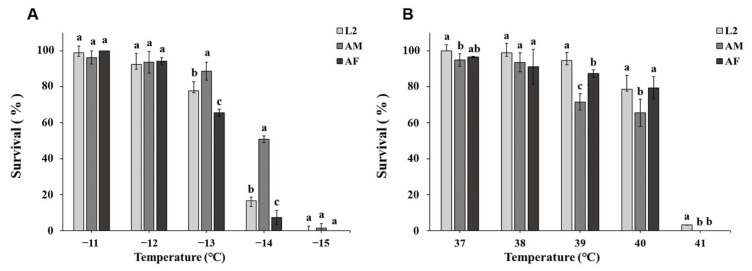
Effect of temperature shock on survival rate of *F. occidentalis* in 2016. Data are denoted as means ± SD. Different lowercase letters indicate significant differences among developmental stages within the same temperature (*p* < 0.05). AF: adult female, AM: adult male, L2: 2nd instar larvae. (**A**) Low-temperature treatments. (**B**) High-temperature treatments.

In P2016, low temperature, sex/stage, and temperature × sex/stage all had significant effects on survival (temperature: *F* = 1068.23, *df* = 4, *p* < 0.001; sex/stage: *F* = 35.48, *df* = 2, *p* < 0.001; temperature × sex/stage: *F* = 23.01, *df* = 8, *p* < 0.001). After exposure to a range of low temperatures (from −11 to −15 °C at 1 °C increments) for 1 h, male survival, especially at −14 °C, was higher than that of females and 2nd instar larvae. The initial lethal low temperature of WFT was −11 °C (−11 °C: *F*_2,6_ = 3.836, *p* = 0.039; −12 °C: *F*_2,6_ = 4.136, *p* = 0.037; −13 °C: *F*_2,6_ = 39.329, *p* < 0.001; −14 °C: *F*_2,6_ = 156.610, *p* < 0.001; −15 °C: *F*_2,6_ = 1.000, *p* = 0.122) (Figure 2A).

**Figure 3:**
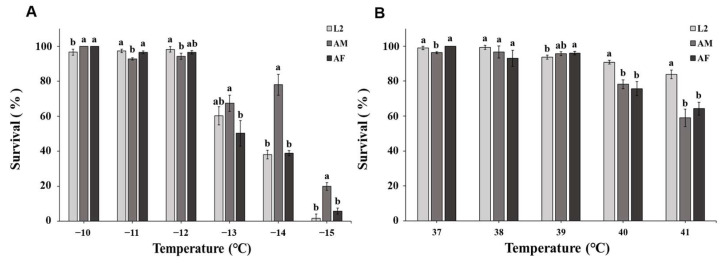
Effect of temperature shock on survival rate of *F. occidentalis* in 2022. Data are denoted as means ± SD. Different lowercase letters indicate significant differences among developmental stages within the same temperature (*p* < 0.05). AF: adult female, AM: adult male, L2: 2nd instar larvae. (**A**) Low-temperature treatments. (**B**) High-temperature treatments.

In P2022, the correlation between factors and survival was similar to that in P2016 (temperature: *F* = 1.170, *df* = 5, *p* < 0.001; sex/stage: *F* = 0.132, *df* = 2, *p* = 0.077; temperature × sex/stage: *F* = 173.422, *df* = 8, *p* < 0.001). At −14 ° C and −15 °C, the male adults showed a very prominent tolerance to cold, as in P2016. The initial lethal low temperature of WFT was −11 °C (−10 °C: *F*_2,6_ = 6.250, *p* = 0.034; −11 °C: *F*_2,6_ = 14.333, *p* = 0.005; −12 °C: *F*_2,6_ = 5.613, *p* = 0.033; −13 °C: *F*_2,6_ = 7.253, *p* = 0.011; −14 °C: *F*_2,6_ = 69.093, *p* < 0.001; −15 °C: *F*_2,6_ =36.897, *p* < 0.001) (Figure 3A).

**Figure 4:**
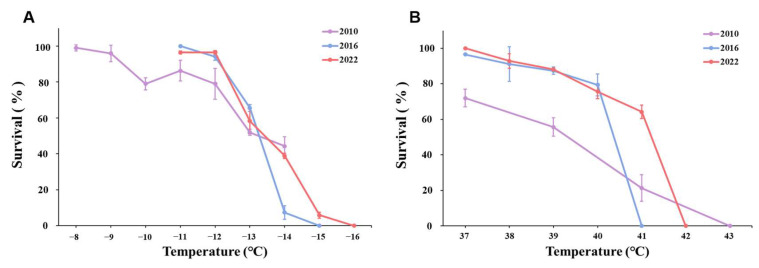
Survival rate comparison of females from the three populations exposed to the same temperature shock. Data are denoted as means ± SD. (**A**) Low-temperature treatments. (**B**) High-temperature treatments.

**Figure 5:**
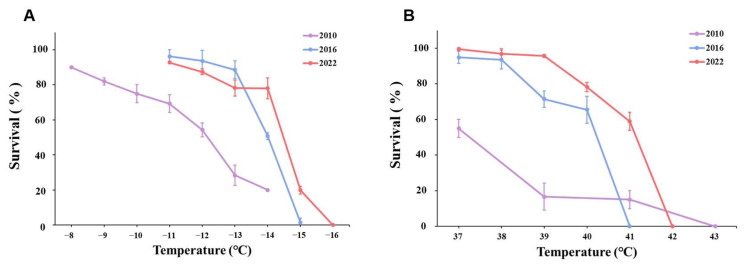
Survival rate comparison of males from the three populations exposed to the same temperature shock. Data are denoted as means ± SD. (**A**) Low-temperature treatments. (**B**) High-temperature treatments.

**Figure 6:**
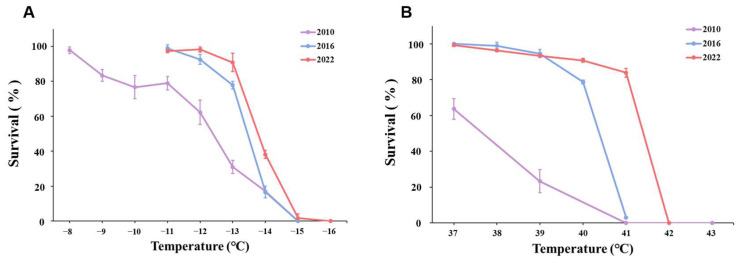
Survival rate comparison of 2nd instar larvae from the three populations exposed to the same temperature shock. Data are denoted as means ± SD. (**A**) Low-temperature treatments. (**B**) High-temperature treatments.

There were significant differences between connected populations, including females, males, and 2nd instar larvae. When the temperature was lower than −13 °C, the survival of P2010 females decreased sharply and was lower than that of P2016 and P2022 females. Although P2010 had a higher survival rate at −14 °C than P2016 and P2022, when the temperature was further reduced to −15 °C, all females in P2010 and P2016 died, while a few females survived in P2022. This same pattern was found in males and 2nd instar larvae. Compared with the P2010 population, adult and 2nd instar larval of the P2016 and P2022 populations showed an increase in cold tolerance, which was reflected by a lower completely lethal temperature or a higher survival rate under the same extreme cold in the acclimatized populations (Figure 4A, Figure 5A and Figure 6A). These results indicated that the cold tolerance of the WFT population increased with the extension of laboratory acclimation time.

LT_50_ values were calculated based on survival rates following cold exposure, and the results of one-way ANOVA showed that LT50 was significantly higher in P2022 compared to P2010, regardless of the sex/stage (AF: *F*_2,6_ = 6.467, *p* = 0.035; AM: *F*_2,6_ = 22.722, *p* = 0.002; L2: *F*_2,6_ = 5.471, *p* = 0.024) (Table 1). In the P2010 population, the LT_50_ for females, males, and 2nd instar larvae was −12.5, −11.5, and −12.8 °C, respectively. In the P2016 population, the LT_50_ values for females, males, and 2nd instar larvae were −13.2, −13.8, and −13.3 °C, respectively. The LT_50_ of females, males, and 2nd instar larvae in the P2022 population after 14 years of laboratory acclimation was −13.4, −13.0, and −13.4 °C, respectively. Adult and 2nd instar larval survival declined with decreasing temperature regardless of temperature acclimation.

### 3.2. Heat Exposure

Significant effects on the survival of P2010 [5] adults and 2nd instar larvae were found for temperature, sex/stage, and the interaction of temperature × sex/stage when WFT were exposed to high temperatures (temperature: *F* = 152.224, *df* = 4, *p* < 0.001; sex/stage: *F* = 44.21, *df* = 2, *p* < 0.001; temperature × sex: *F* = 4.24, *df* = 8, *p* < 0.001). After being exposed to a range of high temperatures (33–41 °C at 2 °C increments) for 1 h, female survival rates were 97, 93, 77, 59, and 24% at 33, 35, 37, 39, and 41 °C, higher than that of both males and 2nd instar larvae. The initial lethal temperature was close to 33 °C (33 °C: *F*_2,6_ = 3.815, *p* < 0.01; 35 °C: *F*_2,6_ = 19.931, *p* = 0.02; 37 °C: *F*_2,6_ = 7.804, *p* = 0.21; 39 °C: *F*_2,6_ = 30.837, *p* = 0.001; 41 °C: *F*_2,6_ = 13.283, *p* = 0.006) (Figure 1B).

For the P2016 group, high temperature, sex/stage, and the interaction of temperature × sex/stage all had a significant effect on the survival of P2016 individuals at high temperatures (temperature: *F* = 441.6, *df* = 4, *p* < 0.001; sex/stage: *F* = 14.67, *df* = 2, *p* < 0.001). There was no significant interaction between temperature and sex (*F* = 1.775, *df* = 8, *p* = 0.107). The survival of females, males, and 2nd instar larvae all had a negative relationship with increasing temperature. When exposed to 40 and 41 °C for 1 h, female survival was 79 and 0%, male survival was 65 and 0%, and that for 2nd instar larva was 79 and 3%. The initial lethal temperature was 37 °C (37 °C: *F*_2,6_ = 5.003, *p* = 0.053; 38 °C: *F*_2,6_ = 4.136, *p* = 0.032; 39 °C: *F*_2,6_ = 41.465, *p* < 0.001; 40 °C: *F*_2,6_ = 5.756, *p* = 0.031; 41 °C: *F*_2,6_ = 552.250, *p* < 0.001) (Figure 2B).

The results of the P2022 experiments showed that high temperature, sex/stage, and the interaction of temperature × sex/status had significant effects on the survival rate of WFT individuals under high temperature (temperature: *F* = 23.710, *df* = 4, *p* < 0.001; sex/stage: *F* = 2.367, *df* = 2, *p* = 0.006; temperature ×sex/stage: *F* = 26.088, *df* = 6, *p* < 0.001). In P2022, the initial lethal temperature of WFT was about 37 °C. When the temperature increased to 39 °C ~40 °C, the survival rate of all sexes/stages decreased significantly. After exposure at 41 °C for 1 h, the survival rates of 2nd instar larvae and male and female adults decreased to 84%, 59%, and 64%, respectively. When the temperature increased from 41 °C to 42 °C, the survival rate of all sex/stage plummeted to 0%. In addition, under the extreme high temperature of 41 °C~42 °C, the survival rate of 2nd instar larvae was higher than that of male and female adults, showing stronger high-temperature resistance than that of male and female adults (37 °C: *F*_2,6_ = 24.250, *p* = 0.001; 38 °C: *F*_2,6_ = 5.625, *p* = 0.032; 39 °C: *F*_2,6_ = 4.909, *p* = 0.042; 40 °C: *F*_2,6_ = 30.516, *p* = 0.001; 41 °C: *F*_2,6_ = 21.571, *p* = 0.002) (Figure 3B).

Similar to with cold exposure, we found significant differences in the effect of heat exposure among the three populations. The female survival of P2010 was lower than that of the P2016 population and the survival of P2016 females was lower than that of P2022 once the temperature approached lethal temperature levels, and a similar pattern was observed in males. P2022 females, males, and 2nd instar larvae were all much more sensitive to high temperature than P2010 and P2016 (Figure 4A, Figure 5A and Figure 6A).

Comparing the LT_50_ of P2010 with that of P2016, we found that LT_50_ values significantly increased by 1.1, 2.8, and 3.3 °C for acclimated females, males, and 2nd instar larvae, respectively. Additionally, the LT_50_ value of P2016 2nd instar larvae also decreased by 0.8 °C compared to P2022, even if there was no significant difference (AF: *F*_2,6_ = 7.559, *p* = 0.021; AM: *F*_2,6_ = 18.490, *p* = 0.003; L2: *F*_2,6_ = 14.694, *p* = 0.005) (Table 2).

## 4. Discussion

In this study, we evaluated the effect of long-term acclimation at constant temperature on the thermal tolerance of WFT by observing thrips’ survival after exposure for one hour to sub-zero or high temperatures. We found that the temperature adaptation of WFT developed with the extension of acclimatized time under constant temperature and humidity in the laboratory.

Cold tolerance is associated with physiological and molecular mechanisms in insects. For instance, Scharf [17] reported that cold tolerance was higher with increased body mass and lipid content. High levels of trehalose and proline have also been found to be correlated with cold tolerance in *Sitophilus granarius* (L.) and *Cryptolestes ferrugineus* (Stephens) [18]. Insect survival is also related to water and ion homeostasis [19], and insects need to maintain ion homeostasis to cope with chilling [20]. Various molecular mechanisms are involved in this process, including heat shock proteins (Hsp) and DNA methylation [21,22]. Cold stress also causes upregulation of genes in the insect immune pathways [23]. Teets and MacMillan proposed that oxidative stress associated with cold exposure may affect mitochondria and regulate a range of immune related events, and that this synergistic interaction could potentially account for the enhanced cold resistance observed in insects [24].

A previous study [5] found that female adults tolerated low temperatures better than male adults and 2nd instar larvae. However, in our study, the female survival was lower than that of males and 2nd instar larvae after long-term acclimation, especially when WFT were exposed to −14 °C. The previous study [5] suggested some reasons for the difference in temperature tolerance between male and female WFT. For example, Ju et al. [25] found that female *Corythucha ciliate* (Say) have a lower supercooling point and higher survival rate than males. In addition to these, the reproductive characteristics of females may play an important role. Such differences in cold tolerance between males and females exist in various species. Male *S. granarius* (L.), for example, were found to be hardier than females due to higher levels of proline, asparagine, and glutamic acid [18].

We also found differences in the cold tolerance of WFT at different developmental stages. In a similar study, the interaction between temperature and development stage affected the egg developmental rate of *Operophtera brumata* (L.) [26]. This suggests that this same interaction has an influence on the survival of WFT adults vs. larvae. Food utilization rate differs with stage, as larvae only need to fuel their own development while adults need to eat enough to reproduce. Environmental stresses such as cold temperatures, as well as developmental stage have been found to regulate the expression of the *Hsp* (heat shock protein) gene [27], and *Hsps* in *F. occidentalis* have been found to have different expression levels at different development stages [28]. A related study found significant differences in the supercooling point and freezing point of different developmental stages [29].

Exposure to high temperature causes water loss in insect bodies, shortened longevity, and decreased fecundity [30]. In this study, we found that whether WFT were acclimatized or not, the heat tolerance of females was stronger than that of males. This same situation in *Grapholita molesta* (Busck) adults was found to correlate with the expression profiles of *Hsp 90* and *Hsp 70* genes [31], and in another study the expression of *Hsp 25.4* was down-regulated in the male fat body of *Bombyx mori* (L.) [32]. Meanwhile, the greater tolerance of WFT females to high temperature may also simply be because they are larger [33]. However, this phenomenon does not exist in all insects, as discussed in Li [34].

Male WFT in particular were the least able to cope with high temperatures, perhaps because they have a smaller body size than either females or 2nd instar larvae. Meanwhile, the heat tolerance of P2022 2nd instar larvae was significantly greater than the larvae of P2010 and P2016. Chidawanyika et al. [35] found that heat tolerance decreased with increasing age and was highest in middle-aged beetles. However, another report found the opposite result, an increase in thermal tolerance with increasing age [36]. These responses may therefore be stress- or species-specific.

Our results demonstrate that long-term laboratory acclimation significantly improved the thermal tolerance of WFT, as reflected by the increased LT50 values in both adults and 2nd instar larvae. While most studies attribute insect adaptation to environmental changes [37,38], we observed enhanced temperature tolerance in WFT populations despite maintaining constant rearing conditions for over a decade. This suggests that environmental fluctuation is not the sole driver of thermal adaptation in this species. Typically, under laboratory conditions, insects are prone to weight loss, fertility decline, prolonged development periods, and even mass mortality after several generations of indoor breeding [39,40]. Among the contributing factors, genetic degradation is the most fundamental cause. The captive populations lack natural selection pressures, and continuous clonal reproduction and inbreeding reduce genetic heterogeneity, leading to increased frequencies of pathogenic genes and other deleterious alleles. This ultimately results in overall fitness decline across multiple traits [41,42]. Notably, unlike typical cases of laboratory population degradation [43,44], WFT populations not only maintained stable fitness without reintroduction but actually evolved greater heat tolerance. We propose that WFT’s unique reproductive strategy—alternating between sexual reproduction and arrhenotoky—provides a mechanistic explanation for this exceptional adaptability. The sexual reproduction phase enables chromosomal recombination and purging of deleterious mutations through natural selection [45], while the arrhenotoky reproductive phase reduces deleterious mutations in haploid males and enhances beneficial allele transmission [15,46]. This reproductive strategy has been widely studied in aphids. Numerous hypotheses have been proposed, including the elimination of deleterious mutations, adaptation to environmental changes, and telomere length changes and reset. However, the underlying mechanisms remain incompletely understood [47]. Although current research on this topic is incomplete, thrip is one of the representative populations of the coexistence of sexual and asexual lineages. Future studies can use thrips as a model to verify and improve some of the relevant conjectures mentioned above, and to address the questions regarding the evolution and adaptation potential of sexual and asexual reproduction.

## 5. Conclusions

Our results demonstrate that long-term laboratory acclimation under constant conditions significantly enhanced thermal tolerance in *Frankliniella occidentalis* populations. Specifically, we observed generational improvements in both cold tolerance (with LT_50_ decreasing by 0.9 °C for females and 1.5 °C for males from P2010 to P2022) and heat tolerance (LT_50_ increasing by 4.1 °C for 2nd instar larvae). The thermal adaptation showed stage- and sex-specific divergence: While females retained superior heat tolerance, they exhibited reduced cold survival at extreme temperatures (−14° C), whereas males demonstrated unexpected cold resilience. This evolution of thermal tolerance contrasts sharply with classical degradation patterns typically observed in laboratory-reared insects, as *F. occidentalis* maintained and even improved fitness without genetic supplementation or environmental variability. We propose that the unique arrhenotoky–sexual reproduction alternation may underlie this adaptation, potentially functioning through two complementary mechanisms: purging deleterious mutations via sexual recombination and enhancing beneficial allele transmission through haploid males. Collectively, these findings challenge the prevailing paradigm that environmental variability is essential for thermal adaptation in insects. Our study provides the first experimental evidence that an intrinsic evolutionary mechanism within *F. occidentalis* populations exists, and establishes a new framework for understanding insect population evolution.

## Figures and Tables

**Table 1:** Lower lethal temperatures (LT_50_) for *F. occidentalis* 2010 [5], 2016, and 2022 populations when exposed to a range of temperatures for an hour.

Developmental Stage	Year	LT_50_ (°C)	95% Confidence Limits
AF	2010	−12.5	−13.3~−12.0 a
	2016	−13.2	−13.4~−12.9 ab
	2022	−13.4	−13.2~−13.6 b
AM	2010	−11.5	−12.2~−10.8 a
	2016	−13.8	−14.2~−13.4 b
	2022	−13.0	−12.6~−13.3 b
L2	2010	−12.8	−11.5~−13.7 a
	2016	−13.3	−13.8~−12.8 b
	2022	−13.4	−12.9~−13.9 b

Different lowercase letters indicate significant differences among the 2010, 2016 and 2022 populations within the same developmental stage (*p* < 0.05). Letters are color-coded by developmental stage: red (AF: adult female), blue (AM: adult male), green (L2: 2nd instar larvae).

**Table 2:** Higher lethal temperatures (LT_50_) for *F. occidentalis* 2010 [5], 2016 and 2022 populations when exposed to a range of temperatures for an hour.

Developmental Stage	Year	LT_50_ (°C)	95% Confidence Limits
AF	2010	38.9	38.3~39.6 a
	2016	40.0	39.5~40.6 a
	2022	39.6	38.7~40.8 a
AM	2010	36.9	35.9~37.7 b
	2016	39.7	39.3~40.1 a
	2022	40.5	39.7~41.6 a
L2	2010	36.9	36.1~38.6 b
	2016	40.2	39.8~40.7 a
	2022	41.0	40.2~41.8 a

Different lowercase letters indicate significant differences among the 2010, 2016 and 2022 populations within the same developmental stage (*p* < 0.05). Letters are color-coded by developmental stage: red (AF: adult female), blue (AM: adult male), green (L2: 2nd instar larvae).

## Data Availability

The raw data supporting the conclusions of this article will be made available by the authors on request.
